# The nesting preference of an invasive ant is associated with the cues produced by actinobacteria in soil

**DOI:** 10.1371/journal.ppat.1008800

**Published:** 2020-09-10

**Authors:** Hongmei Huang, Lu Ren, Huijing Li, Axel Schmidt, Jonathan Gershenzon, Yongyue Lu, Daifeng Cheng

**Affiliations:** 1 Department of Entomology, South China Agricultural University, Guangzhou, China; 2 Department of Biochemistry, Max Planck Institute for Chemical Ecology, Jena, Germany; University of Georgia, UNITED STATES

## Abstract

Soil-dwelling animals are at risk of pathogen infection in soils. When choosing nesting sites, animals could reduce this risk by avoiding contact with pathogens, yet there is currently little evidence. We tested this hypothesis using *Solenopsis invicta* as a model system. Newly mated queens of *S*. *invicta* were found to nest preferentially in soil containing more actinobacteria of *Streptomyces* and *Nocardiopsis* and to be attracted to two volatiles produced by these bacteria, geosmin and 2-methylisoborneol. Actinobacteria-rich soil was favored by *S*. *invicta* and this soil contained fewer putative entomopathogenic fungi than adjacent areas. Queens in such soil benefited from a higher survival rate. In culture, isolated actinobacteria inhibited entomopathogenic fungi, suggested that their presence may reduce the risk of fungal infection. These results indicated a soil-dwelling ant may choose nest sites presenting relatively low pathogen risk by detecting the odors produced by bacteria with anti-fungal properties.

## Author summary

Insect pathogens are widely distributed in soil. Soil-dwelling insects must overcome challenges arising from pathogens in soil. Here we report that a soil-dwelling ant may choose nest sites with lower pathogen infection risk, specifically the ant can sense the cues of some actinobacteria that can inhibit the growth of the pathogens. By choosing the sites with higher abundance of some actinobacteria, the ant can get a higher survival rate. The ant and some actinobacteria thus coordinate a specialized adaptive strategy of infection risk management, enabling the ant population to grow.

## Introduction

The risk of predators, pathogen infection and abiotic condition is a major criterion used by insects when choosing nesting sites [1, 2]. In particular, for social insects that nest in soil, pathogens are a significant danger that varies greatly from place to place [3–6]. Though some insects can detect pathogens and avoid them [7], it remains unclear whether social insects can reliably detect pathogens at a potential nesting site and avoid them. Queens of wood ant were attracted to fungal pathogens during the initial stage of colony founding [8]. Pathogens manipulate uninfected insect hosts deserves further investigation since records of pathogens attracting their hosts are indeed rare, as hosts are under strong selection to resist manipulation and to avoid virulent pathogens [9, 10].

Social insects have highly developed chemical communication systems [11–15] and are sensitive to numerous cues from conspecifics, heterospecifics and the environment that alter their behavior [16–18]. Identifying these cues can facilitate the understanding of the complex behaviors of social insects and the manipulation of their abundance. Studies have shown that many volatile compounds in soil are produced by soil actinobacteria, and the soil actinobacteria can even affect the growth of underground pathogenic fungi and aboveground plants [19–21]. Recent studies indicated that some insects can be attracted or repelled by the geosmin or 2-methylisoborneol produced by the actinobacteria [22–24]. Geosmin induces aversion in *Drosophila melanogaster* presumably signaling the presence of harmful microbes on food [24]. In contrast to flies, geosmin is not aversive but mediated egg-laying site selection in *Aedes aegypti*. [23]. *Streptomyces* bacteria produced geosmin and 2-methylisoborneol can even attract a soil arthropod to promote spore dispersal [22]. Furthermore, the soil actinobacteria may have evolved into the defensive symbionts of soil-dwelling insects. Once associated with an insect, many actinobacteria can protect insects against pathogens by producing some antibiotics [25]. One of the most striking features of actinobacteria is that they can produce antifungal compounds [26]. Actinobacteria with antifungal activity are reported to show high species richness in certain types of soil [27–29]. Because entomopathogenic fungi are also widely distributed in soil [30–33], we hypothesize that some soil-dwelling insects may choose soil types with the highest abundance of actinobacteria for nest building to avoid being infected by some soil-derived entomopathogenic fungi.

The imported red fire ant *Solenopsis invicta* is a notorious invasive pest in many countries [34], where its rapid spread has caused great economic losses and ecological problems [35] and stimulated research to understand the causes of its invasiveness [36–39]. The nesting behavior of *S*. *invicta* was found to be significantly influenced by environmental conditions [40]. Studies have indicated that climate [41], habitat type [42] and soil attributes [43, 44] can affect the distribution of *S*. *invicta*. Almost five years of observations in China showed that colonies of *S*. *invicta* at relatively fine geographical scales are only distributed in certain parts of the available habitat (S1 Fig). Here, we show that soil from the favored nesting and non-favored nesting areas of *S*. *invicta* are not equally attractive to newly mated queens and workers and the possible reasons behind was tested.

## Results

### *Solenopsis invicta* ants are attracted to soil and soil volatiles from their favored nesting areas

**Fig 1:**
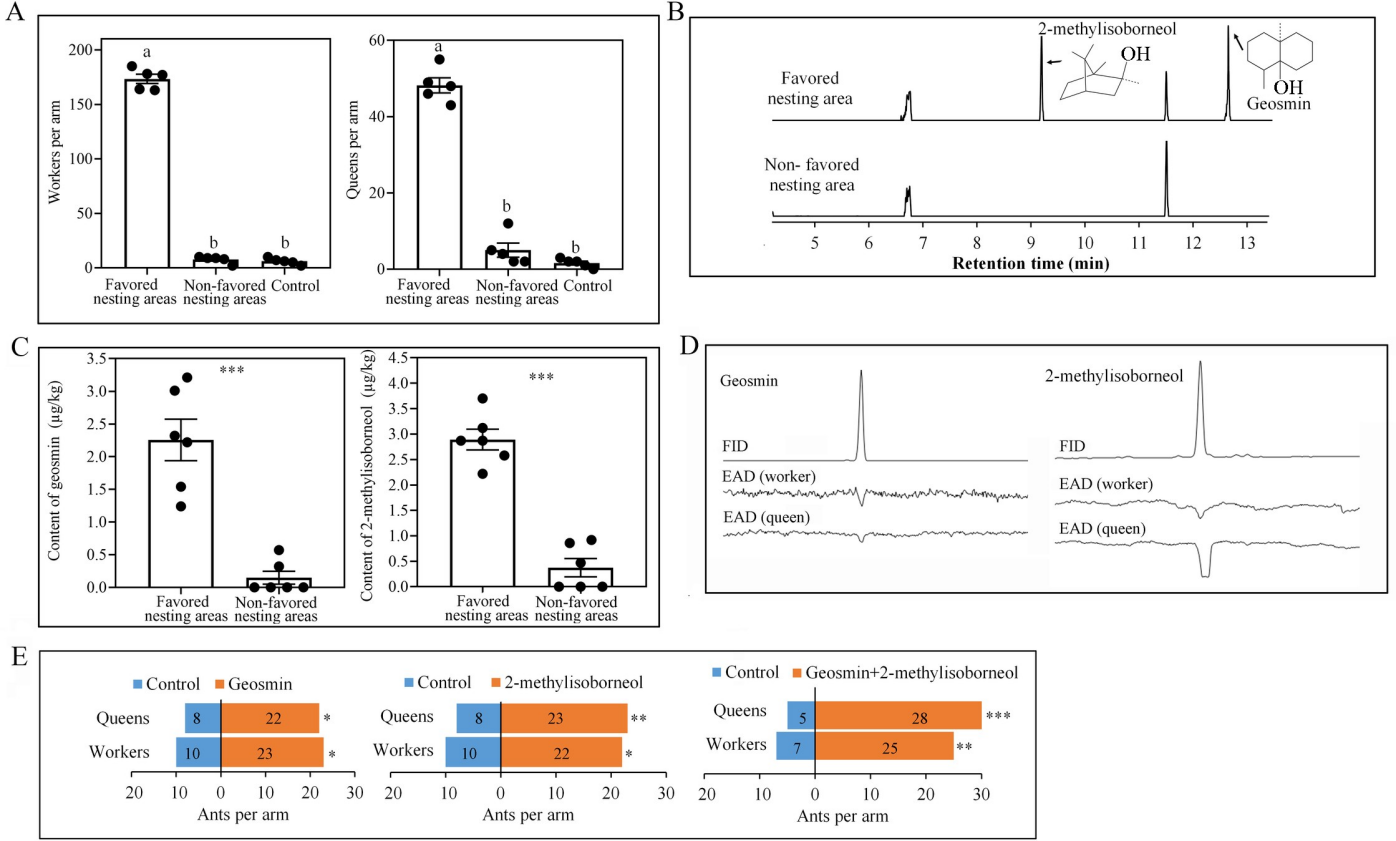
Attraction of *S*. *invicta* to soil from favored nesting areas and to soil volatiles. (A) Choices of workers and queens in regard to soil from favored or non-favored nesting areas in olfactometer bioassays (±SE, *n* = 5 biological replicates). Control: empty arm. (B) Typical gas chromatographic traces obtained from the soil of favored and non-favored nesting areas. (C) Content of geosmin and 2-methylisoborneol (±SE, *n* = 6 biological replicates, independent sample t test). (D) GC−EAD profile of geosmin and 2-methylisoborneol on worker and queen antenna. Upper trace: GC−FID. Lower trace: EAG (unit: mV). (E) Attraction of *S*. *invicta* to geosmin and 2-methylisoborneol in a Y-tube olfactometer (n ≥ 30 biological replicates, chi-square test). Control: mineral oil. Different letters above bars indicate significant differences according to Kendall’s W test at the 0.05 level. Asterisks indicate significant differences (*P < 0.05, **P < 0.01, *** *P* < 0.001).

To determine whether soil from favored nesting areas in the field was more attractive to *S*. *invicta* than soil from non-favored areas, we compared the two soil types in choice assays in an olfactometer. Significantly more workers and queens were recovered from olfactometer arms connected to jars containing soil from favored nesting areas than from arms connected to jars containing soil from non-favored areas (worker: Kendall W = 0.832, χ^2^ = 8.316, *P* = 0.016; queen: Kendall W = 0.84, χ^2^ = 8.4, *P* = 0.015; Fig 1A), indicating that soil from favored nesting areas may release volatile ant attractants. To identify these attractants, soil from favored and non-favored nesting areas was analyzed by GC-MS. A marked difference between the two soils was that the norsesquiterpene geosmin and the monoterpene 2-methylisoborneol were present in soil from favored nesting areas but absent or less from soil from non-favored areas (geosmin: *t* = 6.334, *df* = 10, *P* < 0.01, 2-methylisoborneol: *t* = 9.262, *df* = 10, *P* < 0.01, independent sample t test, Fig 1B and 1C). Moreover, consistent responses of worker and queen antennae were obtained in the GC-EAD analysis (Fig 1D). To test whether geosmin and 2-methylisoborneol were indeed attractive to *S*. *invicta*, authentic standards (Sigma-Aldrich, more than 98% pure) were tested in a Y-tube olfactometer. For this purpose, 2 μg/L geosmin or 2-methylisoborneol were added as the attractants. The results showed that the olfactometer arm containing geosmin or 2-methylisoborneol attracted significantly more ants than the control arm (geosmin: χ^2^ = 5.121, *df* = 1, *P* = 0.024 (worker), χ^2^ = 6.533, *df* = 1, *P* = 0.011 (queen); 2-methylisoborneol: χ^2^ = 4.5, *df* = 1, *P* = 0.034 (worker), χ^2^ = 7.258, *df* = 1, *P* = 0.007 (queen); geosmin + 2-methylisoborneol: χ^2^ = 10.125, *df* = 1, *P* = 0.001 (worker), χ^2^ = 16.03, *df* = 1, *P* < 0.001 (queen), Fig 1E). The concentrations of geosmin and 2-methylisoborneol used in the olfactory bioassays were selected according to the concentrations in soil (Fig 1C), these results indicate that geosmin and 2-methylisoborneol likely attract *S*. *invicta* to its favored nesting areas and should be of ecological relevance.

### Streptomycetaceae and Nocardiaceae are more abundant in soil from favored nesting areas

**Fig 2:**
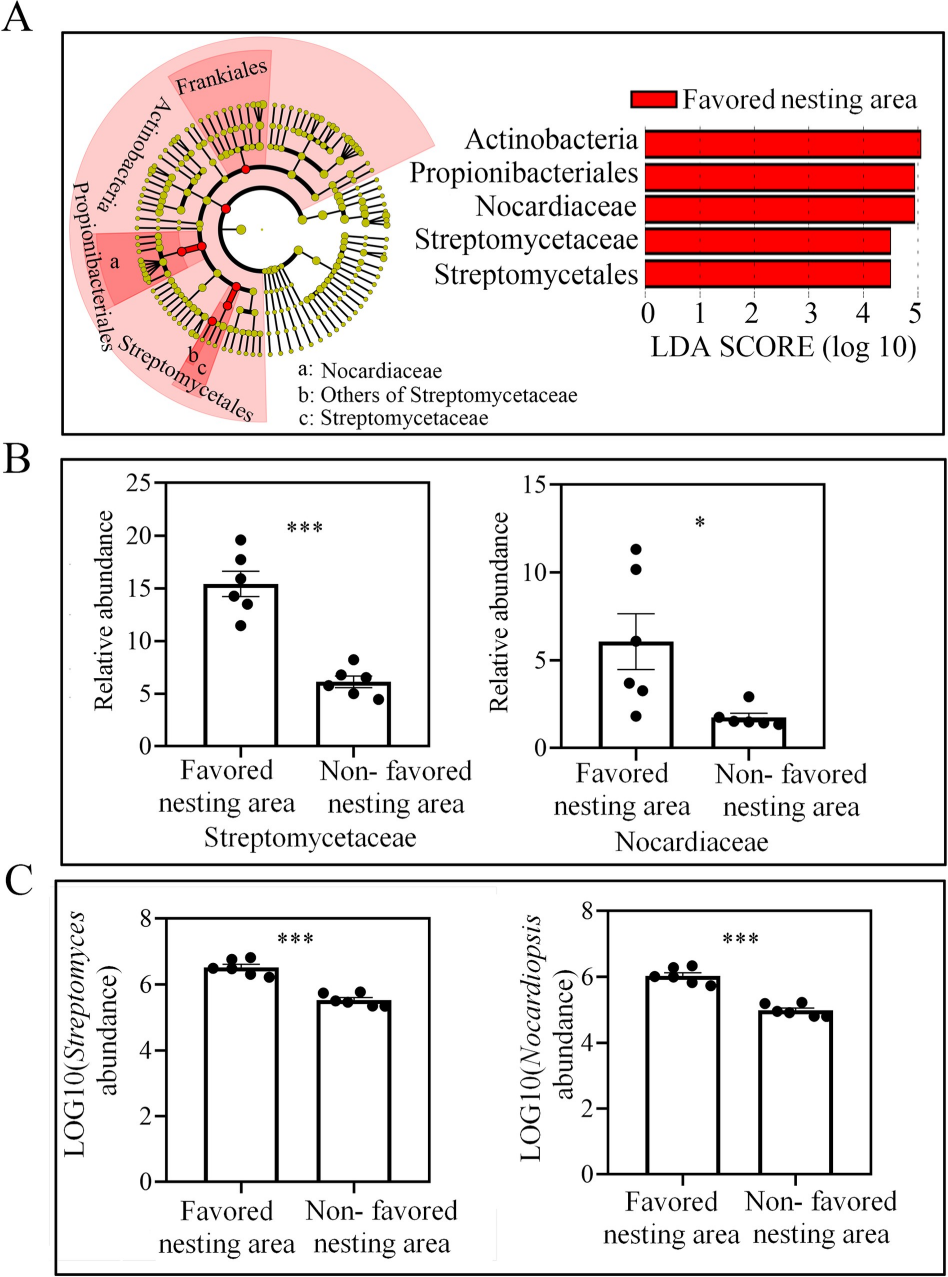
Actinobacterial diversity in soil from favored nesting and non-favored nesting sites. (A) Taxonomic groups responsible for the differences in microbial diversity between favored and non-favored nesting soil based on LEfSe analysis. (B) Relative abundance of Streptomycetaceae and Nocardiaceae in soil from favored vs. non-favored nesting areas (±SE, *n* = 6 biological replicates, independent sample t test). (C) Absolute abundance of *Streptomyces* and *Nocardiopsis* in soil achieved by qPCR (±SE, *n* = 6 biological replicates, independent sample t test). Asterisks indicate significant differences (*P < 0.05, ***P < 0.001).

Studies have indicated that geosmin and 2-methylisoborneol can be produced as secondary metabolites by microorganisms including actinomycetes, particularly *Streptomyces* [45], cyanobacteria [46], myxobacteria [47], fungi [48], and even by some plants [49]. Moreover, some studies have reported that actinomycetes play an important role in the life of ants and other insects [25, 50]. Thus, we hypothesized that the presence of geosmin and 2-methylisoborneol in soil from favored nesting areas may be due to actinobacteria. Thus, the bacterial diversity in soil from favored and non-favored nesting areas was compared with 16S rRNA gene sequencing. The sequencing results indicate that there was no difference between the soils in terms of bacteria operational taxonomic units (OTU) numbers and Shannon diversity index (OTU number: *t* = 0.989, *df* = 10, *P* = 0.346; Bacteria Shannon index: *t* = 1.498, *df* = 10, *P* = 0.165, independent sample t test, S2A Fig and S2B Fig). However, comparison of the Shannon diversity index values for the actinobacteria indicated that the soil samples from favored nesting areas had a significantly lower diversity (*t* = 3.429, *df* = 10, *P* = 0.006, independent sample t test, S2C Fig). A separation of the samples from favored and non-favored nesting areas was also shown by the principal co-ordinates analysis (PCoA) (S2D Fig). We used the LDA Effect Size (LEfSe) method to identify bacterial OTUs that were likely to explain the major differences between the nesting and non-nesting areas. As a result, Streptomycetaceae and Nocardiaceae were identified as biomarkers of the favored nesting sites in the soil (LDA scores > 4.5) (Fig 2A), with significantly higher abundance in soil from favored vs. non-favored areas (Streptomycetaceae: *t* = 6.994, *df* = 10, *P* < 0.01; Nocardiaceae: *t* = 2.68, *df* = 10, *P* = 0.023, independent sample t test, Fig 2B). Because there was no difference in the absolute content of bacteria in soil from favored and non-favored nesting areas (*t* = 0.211, *df* = 10, *P* = 0.837, independent sample t test, S2E Fig), the content of Streptomycetaceae and Nocardiaceae may contribute to the differences between the two soil types. Moreover, significantly higher absolute abundances of *Streptomyces* and *Nocardiopsis* were also detected in favored soil by quantitative PCR (*Streptomyces*: *t* = 7.942, *df* = 10, *P* < 0.01; *Nocardiopsis*: *t* = 8.474, *df* = 10, *P* < 0.01, independent sample t test, Fig 2C).

### Actinobacteria release attractive soil volatiles

**Fig 3:**
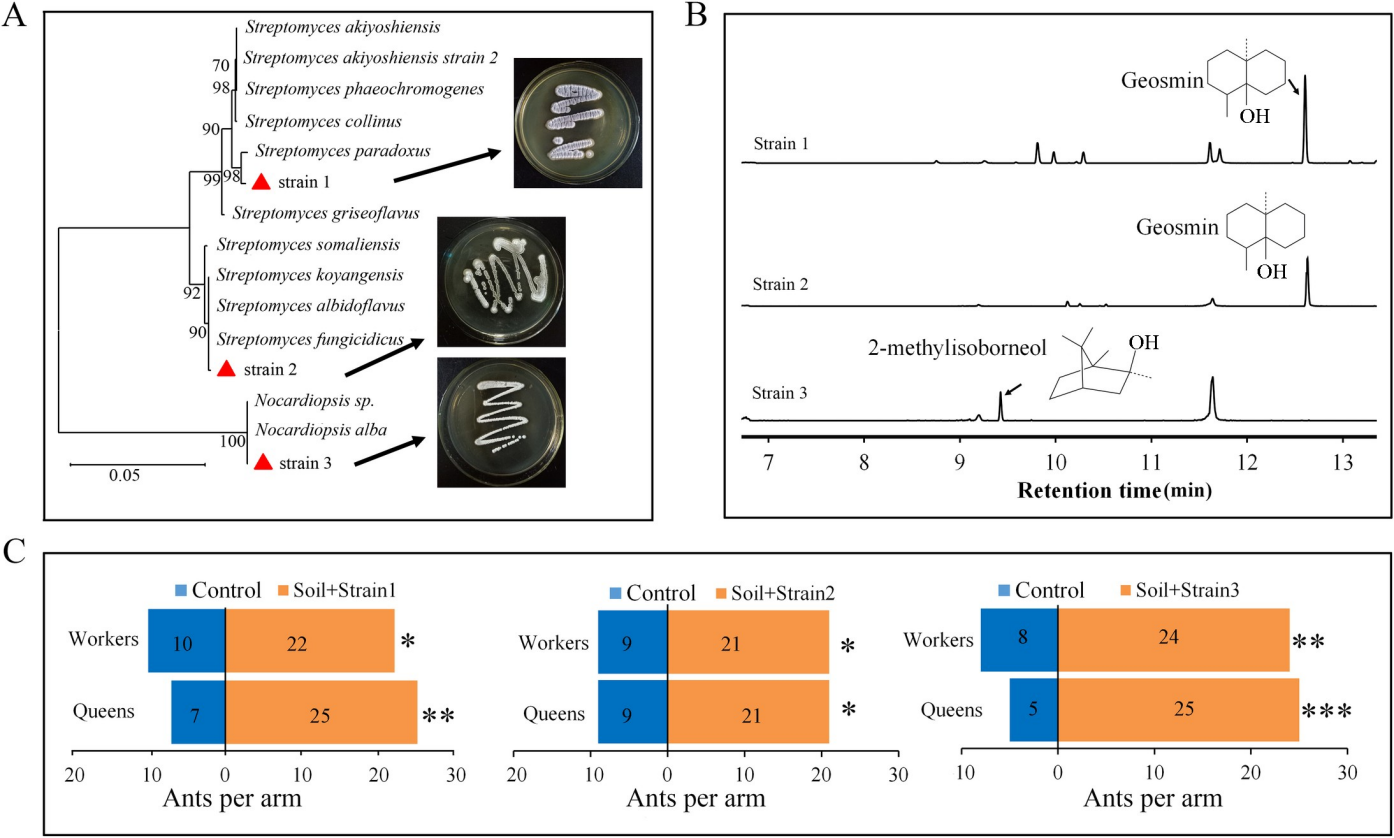
Volatiles of actinobacterial strains attract *S*. *invicta* ants. (A) Molecular phylogenetic analysis of actinobacterial strains isolated from soil from favored nesting sites and identified based on their 16S rRNA gene sequences. Tree was constructed with a maximum likelihood estimation method. Branch support is indicated as bootstrap values (500 replicates). Red triangles indicate the isolated bacteria. (B) Typical gas chromatographic traces of the headspace of isolated bacterial cultures showing the identified volatiles. (C) Attraction of *S*. *invicta* in a Y-tube olfactometer to the isolated bacteria when added to soil from non-favored nesting areas (n ≥ 30 biological replicates, chi-square test). Control: soil from non-favored nesting areas. Asterisks indicate significant differences (*P < 0.05, **P < 0.01, ***P < 0.001).

To verify whether geosmin and 2-methylisoborneol were produced by bacteria of Streptomycetaceae and Nocardiaceae, the bacterial strains of these families were isolated. With morphological identification and 16S rRNA sequencing, only two geosmin producing strains (strain 1 and strain 2) identified as *Streptomyces* and one 2-methylisoborneol producing strain (strain 3) identified as *Nocardiopsis* (99% 16S rRNA sequence similarity) were isolated from the soil samples collected from favored nesting area (Fig 3A and 3B). Volatiles produced by the isolated bacteria in culture were collected by solid-phase microextraction (SPME) and then analyzed with GC-MS. Geosmin was identified as a volatile produced by strain 1 and strain 2, and 2-methylisoborneol was identified as a volatile associated with strain 3 (Fig 3B). Moreover, the number of ants attracted to the olfactometer arms loaded with soil from non-favored nesting areas significantly increased after adding the isolated bacteria (strain 1: χ^2^ = 4.5, *df* = 1, *P* = 0.034 (worker), χ^2^ = 10.125, *df* = 1, *P* = 0.001 (queen); strain 2: χ^2^ = 4.8, *df* = 1, *P* = 0.028 (worker), χ^2^ = 4.8, *df* = 1, *P* = 0.028 (queen); strain 3: χ^2^ = 8, *df* = 1, *P* = 0.005 (worker), χ^2^ = 13.33, *df* = 1, *P* < 0.001 (queen), Fig 3C).

### Pathogenic fungi was less abundant in soil from favored nesting areas

**Fig 4:**
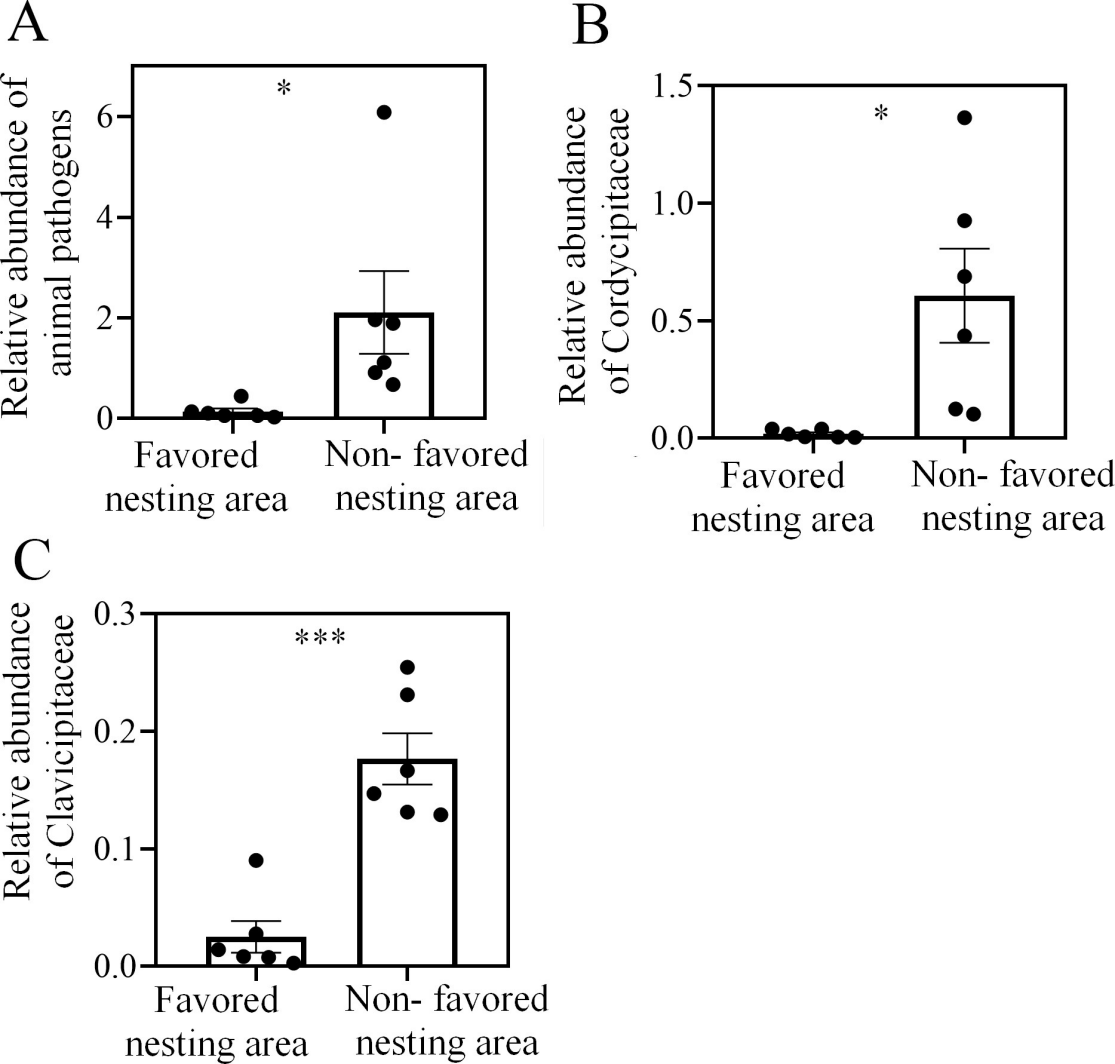
Diversity of fungi is greater in soil from areas not favored by nesting *S*. *invicta* queens. (A) Relative abundance of fungi that are pathogenic to animals (±SE, *n* = 6 biological replicates, independent-sample t tests). (B) and (C) Relative abundance of Clavicipitaceae and Cordycipitaceae (±SE, *n* = 6 biological replicates, independent-sample t tests). Asterisks indicate significant differences (*P < 0.05, **P < 0.01, ***P < 0.001).

To test whether *S*. *invicta* queens choose soil with a high abundance of actinobacteria for nest building is aim to avoid being infected by soil-derived entomopathogenic fungi, fungal diversity in *S*. *invicta*-favored and non-favored soil was compared by ITS gene sequencing. There was no difference in OTU number between the favored and non-favored soil (*t* = 1.717, *df* = 10, *P* = 0.117, independent sample t test, S3A Fig), but the soil samples from favored nesting areas had lower Shannon diversity index values (*t* = 2.916, *df* = 10, *P* = 0.015, independent sample t test, S3B Fig). The PCoA also showed a separation of samples from favored and non-favored nesting areas (S3C Fig). FUNGulid (Fungi Functional Guild) classification and annotation revealed that the total relative abundance (proportion of sequencing tags in the total sequencing tags) of OTUs representing taxa that are pathogenic to insects and other animals (mainly Clavicipitaceae (*Metarhizium* sp.) and Cordycipitaceae (*Beauveria* sp.) S1 Data) was significantly lower in soil from the favored nesting areas (animal pathogen: *t* = 2.384, *df* = 10, *P* = 0.038; Clavicipitaceae: *t* = 5.899, *df* = 10, *P* < 0.001; Cordycipitaceae: *t* = 2.943, *df* = 10, *P* = 0.015, independent sample t test, Fig 4). Given that there was no significant difference in total fungal content between the two areas (*t* = 0.563, *df* = 10, *P* = 0.586, independent sample t test, S3D Fig), the absolute number of strains pathogenic to animals was likely lower in soil from the favored nesting area.

### Actinobacteria can increase survival of the queens

**Fig 5:**
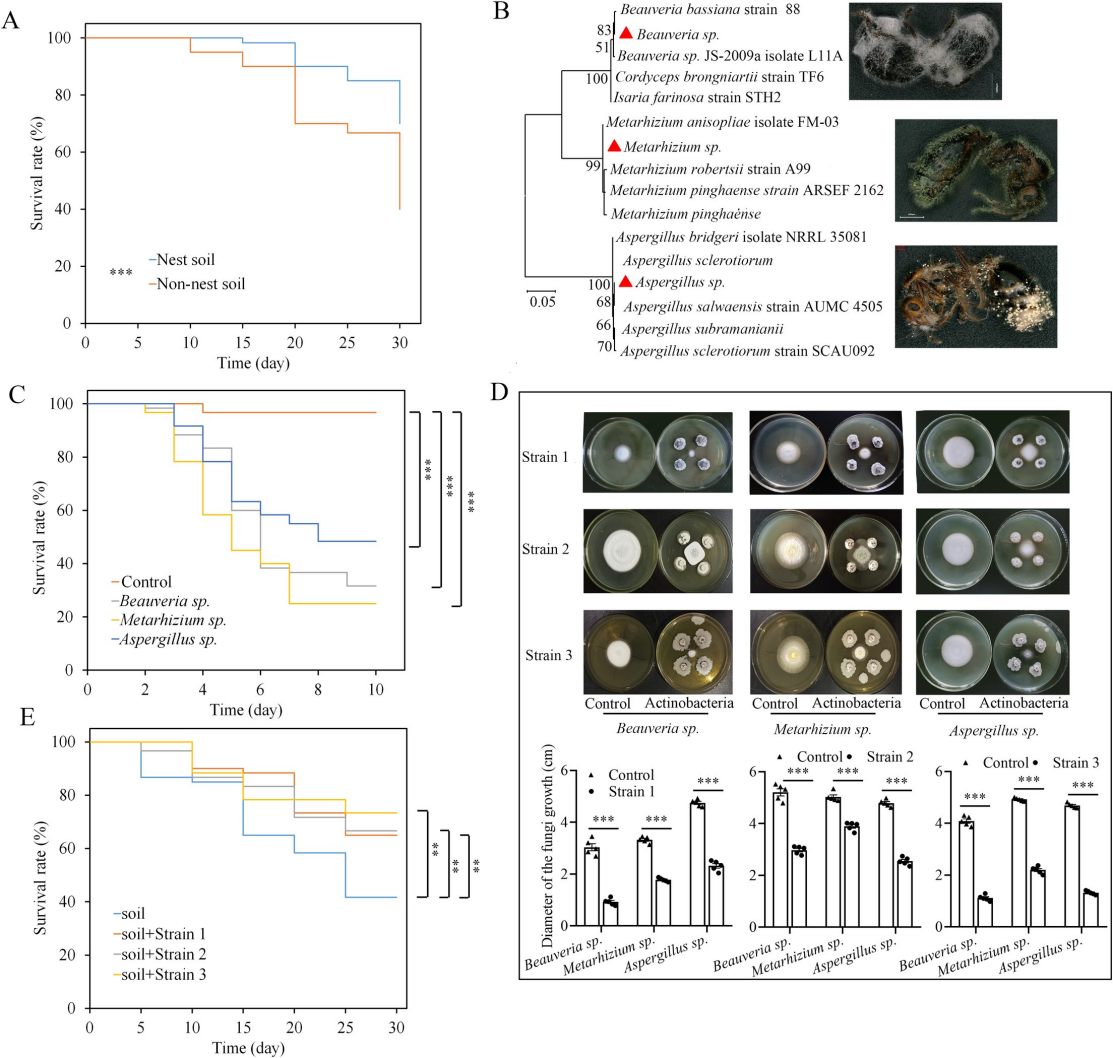
Entomopathogenic fungi from soil not favored by *S*. *invicta* decreases the survival of ant queens. (A) Kaplan-Meier plot of queen survival after they were forced to build nests in soil from nesting and non-nesting sites (log-rank test). (B) Molecular phylogenetic analysis of fungal strains isolated from soil from non-favored nesting areas based on ITS gene sequences. Tree was constructed with a maximum likelihood estimation method. Branch support is indicated as bootstrap values (500 replicates). Red triangles indicate the isolated fungi. (C) Kaplan-Meier plot of *S*. *invicta* queen survival after infection with the isolated fungi (log-rank test). (D) Inhibition effects of the isolated actinobacteria (Fig 3) on the entomopathogenic fungi. On each plate, the fungi were placed in the middle, and the actinobacteria were placed on the four corners of the fungal plug. (E) Kaplan-Meier plot of queen survival after adding the isolated strains of actinobacteria to soil from non-favored nesting sites (log-rank test). Asterisks indicate significant differences (*P < 0.05, **P < 0.01, ***P < 0.001).

To determine whether the increased number of pathogenic fungi had any effect on *S*. *invicta* ants, the survival of newly mated queens was compared in soil from favored vs. non-favored nesting sites. Survival was significantly greater when queens built nests in soil from favored nesting areas (χ2 = 10.975, *df* = 1, *P* = 0.001, Kaplan-Meier plot, log-rank test, Fig 5A). When the dead queens from non-favored soil were collected and placed on wet filter papers, fungi grew out of their bodies after 7 days. With ITS gene sequencing, the fungi were identified as *Aspergillus* sp., *Beauveria* sp. and *Metarhizium* sp. (99% ITS gene sequence similarity) (Fig 5B). The numbers of queens infected by *Aspergillus* sp., *Beauveria* sp. and *Metarhizium* sp. were 6, 18 and 12, respectively. Though, *Aspergillus sp*. was not classified as “pathogenic”, reinfecting new queens with this fungi showed that it can significantly decrease the survival rate as *Beauveria* sp. and *Metarhizium* sp. did (*Aspergillus sp*. vs control: χ^2^ = 34.856, *df* = 1, *P* < 0.001; *Beauveria* sp. vs control: χ^2^ = 54.349, *df* = 1, *P* < 0.001; *Metarhizium* sp. vs control: χ^2^ = 66.243, *df* = 1, *P* < 0.001, Kaplan-Meier plot, log-rank test, Fig 5C). And no fungi were isolated from dead ants from favored soils or the fungi were saprophytic fungi. These results indicate that putative entomopathogenic fungi in the soil from non-favored nesting areas may be one reason that causes death of queens in soil. To test whether the isolated actinobacteria contributed to the lower abundance of entomopathogenic fungi in soil from favored nesting areas, we tested the antifungal activities of the previously isolated actinobacterial strains. All of the isolated actinobacteria had the ability to inhibit the growth of the isolated entomopathogenic fungi, the diameters of the fungi growth are significantly smaller than the ones in controls (Fig 5D). Moreover, the survival rate of the ant queens could be significantly increased by adding the isolated actinobacteria to the soil from non-favored nesting areas (strain 1: χ^2^ = 7.061, *df* = 1, *P* = 0.008; strain 2: χ^2^ = 6.836, *df* = 1, *P* = 0.009; strain 3: χ^2^ = 11.024, *df* = 1, *P* = 0.001, Kaplan-Meier plot, log-rank test, Fig 5E). Moreover, in soil added strain 1, the numbers of dead queens infected by *Aspergillus* sp., *Beauveria* sp. and *Metarhizium* sp. were 0, 7 and 5, respectively; in soil added strain 2, the numbers of dead queens infected by these fungi. were 1, 3 and 6, respectively; in soil added strain 3, the numbers of dead queens infected by these fungi were 0, 4 and 1, respectively; while the numbers of dead queens (in control) infected by *Aspergillus* sp., *Beauveria* sp. and *Metarhizium* sp. were 4, 15 and 14, respectively. These results indicate that actinobacteria may increase the survival of *S*. *invicta* queens by inhibiting the growth of putative entomopathogenic fungi in the soil.

### Data from additional sites further confirmed nesting preference of *S*. *invicta*

**Fig 6:**
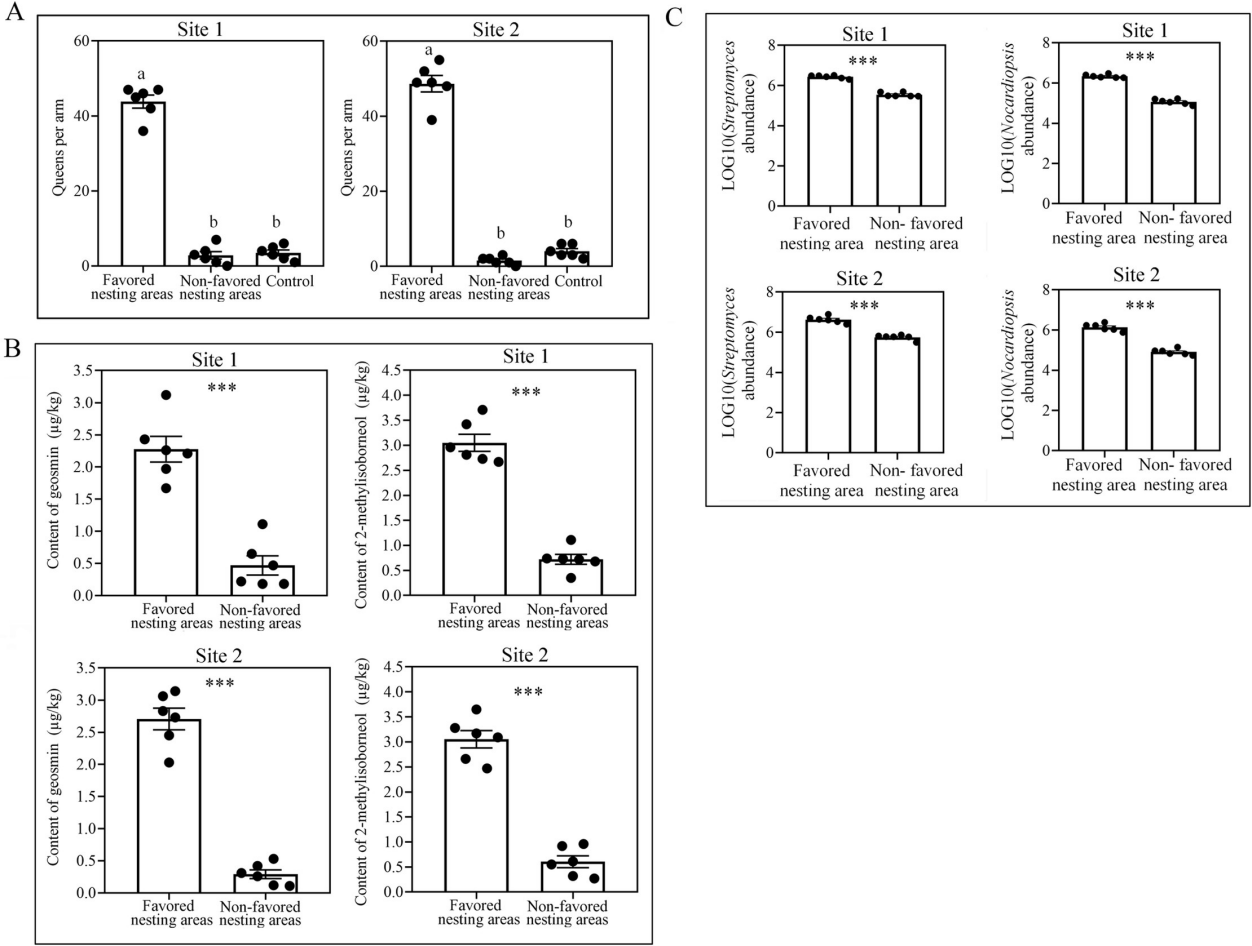
Data from more wild sites confirmed the association between *S*. *invicta* queen nesting preference and actinobacteria. (A) Choices of queens in regard to soil in olfactometer bioassays (±SE, *n* = 6 biological replicates). Control: empty arm. (B) Content of geosmin and 2-methylisoborneol (±SE, *n* = 6 biological replicates, independent sample t test). (C) Absolute abundance of *Streptomyces* and *Nocardiopsis* in soil achieved by qPCR (±SE, *n* = 6 biological replicates, independent sample t test). Different letters above bars indicate significant differences according to Kendall’s W test at the 0.05 level. Asterisks indicate significant differences (*P < 0.05, ***P < 0.001).

The nesting preference of *S*. *invicta* was assessed for another two field sites that are more distant from each other than the core study sites. These additional field data showed that queens preferentially selected the soil from nesting area for the two selected sites (site 1: Kendall W = 0.75, χ^2^ = 9, *P* = 0.011; site 2: Kendall W = 0.964, χ^2^ = 11.565, *P* = 0.003; Fig 6A). Geosmin and 2-methylisoborneol contents were significantly higher in soil from favored nesting areas in the two selected sites (site 1, geosmin: *t* = 7.235, *df* = 10, *P* < 0.001, 2-methylisoborneol: *t* = 11.759, *df* = 10, *P* < 0.001; site 2, geosmin: *t* = 13.299, *df* = 10, *P* < 0.001, 2-methylisoborneol: *t* = 11.589, *df* = 10, *P* < 0.001, independent sample t test, Fig 6B). qPCR assays also revealed that the abundances of *Streptomyces* and *Nocardiopsis* were significantly higher in soil from nesting area for the two selected sites (site 1, *Streptomyces*: *t* = 16.133, *df* = 10, *P* < 0.001; *Nocardiopsis*: *t* = 19.424, *df* = 10, *P* < 0.001; site 2, *Streptomyces*: *t* = 10.19, *df* = 10, *P* < 0.001; *Nocardiopsis*: *t* = 12.915, *df* = 10, *P* < 0.001, independent sample t test, Fig 6C).

## Discussion

In the past century, the red fire ant (*S*. *invicta*) has carried out global invasions with astonishing speed [34] and has become a dominant ant pest in many areas, causing great economic losses and ecological problems [35]. Since 2000, this species has spread to the Caribbean, Australia, New Zealand, Taiwan, Hong Kong, Macao, and China [35]. It can displace native fauna, reduce biodiversity, serve as a vector of pathogens, and impair ecosystem services [51]. Queens can fly considerable distances during mating flights [52], and many factors that affect the movement of queens have been elucidated [53, 54]. However, to the best of our knowledge, this is the first time that the chemical signals of soil microorganisms were reported to affect the nesting choice of newly mated queens. Our findings described the biochemical interactions between microorganisms and insects by microbial volatile organic compound (MVOC) production, which was often overlooked.

There are numerous instances of MVOCs being closely associated with insect feeding behaviors [55], aggregations [56], mating behaviors [57] and oviposition [58]. Emissions from microorganisms *in situ* may even help insect to evaluate habitat suitability or potential exposure to entomopathogens [24], however, such studies are rare and insects were repelled by MVOCs in almost all cases [59]. In contrast to this notion, our study provided a case in another way for which ant was attracted by MVOCs to select suitable habitats. The result provided considerable insight into the evolution of insect behavioral responses to MVOCs.

We demonstrated that queens of *S*. *invicta* are attracted to the volatiles emitted by actinobacteria and to soil from areas colonized by actinobacteria. The areas presented with more actinobacteria may harbor fewer putative entomopathogenic fungi. The newly mated queens are most vulnerable before the first workers emerge. Without the care of workers, queens can easily be infected by pathogens [60]. From an ecological point of view, finding a place to nest that provides protection is very important for the queen's survival. In addition, mating flights occur on sunny days (with a temperature of approximately 26°C) and are most frequent one or two days after rainy days [60]. Meanwhile, the earthy odor produced by geosmin and 2-methylisoborneol can be easily sensed at the mating flight time [61, 62]. Therefore, there may be an ecological correlation between the timing of mating flight and the release of geosmin and 2-methylisoborneol.

The factors that affect the distribution and abundance of ants have attracted wide attention from biologists during recent decades [63]. Studies have indicated that many factors may affect the demography of *S*. *invicta*. For example, climate is believed to be important in limiting fire ant expansion, but its impact at finer geographical scales is more equivocal [41]. Studies have shown that forests often have low fire ant densities, and *S*. *invicta* seems to prefer mesic habitats with high water tables over more xeric sites [42]. Russell et al. (2001) observed more fire ant nests on roadbeds than in adjacent pastures [64]. However, we observed the opposite distributional patterns at the site in our experiments. More mounds are centered in the xeric grassland than on the roadbed (S1 Fig). More generally, ant abundance also appears to be affected by soil attributes [43, 44]. Many studies have shown the effects of soil physical properties on the distribution of ants, while the effects of soil chemistry on *S*. *invicta* abundance have not yet been investigated. Our study investigated the effects of soil chemistry on the distribution of *S*. *invicta*. Our results indicate that soil volatiles produced by actinobacteria may also be one factor that affects the distribution of *S*. *invicta* at fine geographical scales. In fact, the chemical and physical properties of soil may affect one another. Ants may select a certain type of soil for nesting by sensing the chemical cues in the soil. Consistently, research has indicated that the distribution of actinobacteria can be influenced by environmental conditions, such as temperature, moisture, soil type and seasons [65]. Thus, some actinobacteria in soil may be one of the factors that can affect nesting site choice of *S*. *invicta*.

Our results showed for the first time that ants may be attracted to soil based on chemical cues produced by resident actinobacteria, which may provide protection against entomopathogenic fungi. Our finding suggested *S*. *invicta* spread at fine geographical scales may be associated with a potential factor, soil actinobacteria. These findings should prompt new studies into the ecological interactions of insects and soil microbes and the influence of microorganisms on insect distributions. And this could contribute to the management of *S*. *invicta* invasion at fine geographical scales by altering the soil microbiota.

## Materials and Methods

### Sampling

Sample collections were carried out in a residential area in Guangzhou, China (N23.17594389034622; E113.3647156441803). Soil samples were collected below the 15 cm of surface from both favored and non-favored nesting areas (S1 Fig). Specifically, the soil samples were collected nearby the nests in the favored nesting areas. Newly mated queens and workers were collected from the sampling site in 2017 or 2019. Briefly, newly mated queens were collected once they landed on the cement floor. The collected queens were used for experiments immediately.

### Olfactometer bioassays of the soil samples

The attraction of workers and queens to volatiles from different soils was tested in an olfactometer [66] consisting of a central glass chamber (30 cm in diameter, 30 cm deep) with three equally distributed side arms. These arms connected the central chamber to three glass jars (20 cm in diameter, 20 cm deep, and containing 50 g soil) in which different samples of soils. Fresh air that had passed through an air filter with charcoal was pumped through each of the glass jars at a rate of 1.3 L/min. Five such olfactometers were prepared on a given day. Approximately 200 workers or 60 queens were released in the center of each olfactometer, from where they were free to enter the arms. One day after release, the olfactometer was disassembled, and the numbers of workers or queens in each arm were counted. Five biological replicates were performed. After each replicate, the soil in the jar was replaced with new sampled soil.

### Volatile analysis

The volatile organic compounds present in the soil samples were separated by the stripping technique as described previously [67] and subsequently analyzed by gas chromatography-mass spectrometry (GC-MS). Briefly, 2 kg of soil was collected in a 4 L Erlenmeyer flask, which served as a stripping vessel. A closed stripping system was used to concentrate the volatiles on a 100-μm polydimethylsiloxane (PDMS) SPME fiber (Supelco). After 1 h of stripping, the fiber was removed, and the volatiles were analyzed by MS after thermal desorption, transfered by helium into a gas chromatograph and separation. To determine the contents of geosmin and 2-methylisoborneol in soil, a standard curve was generated with the authentic standards. The standard curves were prepared in triplicate (n = 3) using 0.02, 0.2, and 2, 20 and 200 μg/L solutions of geosmin or 2-methylisoborneol (diluted in mineral oil). And the differences of geosmin and 2-methylisoborneol in soil from two areas were compared with independent sample t test. For the analysis of volatiles from the culture media of the actinobacteria, a 100-μm polydimethylsiloxane (PDMS) SPME fiber (Supelco) was used. Compounds were analyzed by GC-MS with an Agilent 7890B Series GC system coupled to a quadrupole-type-mass-selective detector (Agilent 5977B; transfer line 230°C, source 230°C, ionization potential 70 eV). For actinobacteria volatiles, the fiber was inserted manually into the injector port (250°C) for desorption, and the volatiles were chromatographed on an HP-5 MS column (30 m, 0.25 mm internal diameter, 0.25 μm film thickness). Helium at a constant pressure of 110.9 kPa was used for carrier gas flow. After fiber insertion, the column temperature was maintained at 40°C for 2 min and then increased to 250°C at 10°C/min and maintained for 5 min. After using the full scan mode at a range of 50–300 m/z, geosmin and 2-methylisoborneol were quantified according to their main characteristic ions, 112 m/z and 95 m/z. Geosmin and 2-methylisoborneol identification was based on the comparison of their mass spectra with those listed in the NIST mass spectral library. Additionally, the identification of geosmin and 2-methylisoborneol was confirmed by comparing their retention time and mass spectra with those of authentic standards purchased from suppliers. All soil samples collected from 12 sampling sites in S1 Fig were sent for detecting geosmin and 2-methylisoborneol.

### GC-EAD analysis

To determine whether geosmin and 2-methylisoborneol could elicit ant antennal responses, the GC-EAD analysis was conducted using a gas chromatograph (GC) (6890N, Agilent Technologies) equipped with a flame ionization detector (FID) and an electroantennographic detector (Syntech, Hilversum, Germany). The GC was equipped with a fused silica capillary column (30 cm ×0.25μm) coated with (5%-phenyl) dimethypolysiloxane (HP-5, 0.25μm thick, Agilent Technologies). One microliter of geosmin and 2-methylisoborneol (2μg/L) was injected. The dissected antenna was immediately attached with electrode gel (Spectra 360 Electrode Gel, Parker Laboratories, Orange, NJ) to a metal electrode with 10 × amplification (Syntech). The signal was processed through a two-channel serial-bus acquisition controller (IDAC2, Syntech) and analyzed with software (EAD ver. 2.5 Syntech). For each volatile, electroantennograms were recorded from three antennae cut from three workers or queens. Antennal responses were matched with FID signals of compounds eluting from the GC.

### Olfactometer bioassays of the identified volatiles and actinobacteria

An olfactometer consisted of a Y-shaped glass tube, with a main arm (20 cm length*5 cm diameter) and two lateral arms (20 cm length,5 cm diameter) was used. The lateral arms were connected to glass chambers (20 cm diameter,45 cm height) in which the odor sources were placed. To ensure a supply of odor-free air, both arms of the olfactometer received charcoal-purified and humidified air at a rate of 1.3 L/min.

To test the attraction effect of geosmin, 2-methylisoborneol or mixture of geosmin and 2-methylisoborneol to workers or queens, 1 mL geosmin (2 μg/L diluted in mineral oil), 2-methylisoborneol (2 μg/L diluted in mineral oil) or mixture of geosmin and 2-methylisoborneol (2 μg/L diluted in mineral oil, both geosmin and 2-methylisoborneol have 2 μg in 1 L mineral oil) was placed in one odor glass chamber. In the control odor glass chamber, 1 mL mineral oil was placed. Then Newly mated queens or workers were individually released at the base of the olfactometer and allowed 5 min to show a selective response. The response was recorded when a queen/worker moved into one arm more than 3 cm and stayed for >1 min. Queens or workers that did not leave the base of the olfactometer were recorded as non-responders. Only insects that responded were included in the data analysis (for all experiments, <5% were non-responders). Odor sources were randomly placed in one arm or the other at the beginning of the bioassay and experiment was repeated ten time. The system was washed with alcohol after every experiment. More than 30 queens or workers were selected for testing and each ant was used only once for each odor.

To test the attraction effect of soil from non-favored area (sterilized before using) supplemented with the culture media of the isolated actinobacteria on workers or queens, 200 g soil mixed with 10 ml culture media of the isolated actinobacteria was placed in one odor glass chamber. In the control odor glass chamber, 200 g soil mixed with 10 mL sterilized water was placed. The culture media was prepared as following: square plugs of actinobacteria (0.6 cm on one side) were placed in the middle of the plates (Gauze’s Medium No.1), and the plates were cultured for two weeks. Then, 10 mL of sterile water was used to wash off the bacteria, which were evenly added to 200 g of soil collected from the non-favored nesting area. Then the selective behavior of workers or queens were tested as described above. Odor sources were randomly placed in one arm or the other at the beginning of the bioassay. After 10 replicates, the system was washed with alcohol. For each odor, more than 30 queens or workers were selected for testing and each ant was used only once.

### Microbial diversity analysis

Microbial DNA was extracted from 5 g soil samples using the DNA extraction Kit (Omega Biotek, Norcross, GA, U.S.) according to the manufacturer’s protocols. Before sequencing, qPCR was used to estimate the differences in the absolute abundance of microorganisms. For bacteria, primers (338F: 5’-ACTCCTACGGGAGGCAGCAG-3’; 518R: 5’-ATTACCGCGGCTGCTGG-3’) [68] targeting the 16S rRNA gene were prepared, and the genomic DNA of *E*. *coli* was extracted for amplification with the primers. The amplified fragment was then cloned into the pMD 18–T vector, which was then transferred into *E*. *coli* DH5α to reproduce. The reproduced vector was then extracted with a plasmid extraction kit, and diluted in a series of 10-fold dilutions to obtain 5 different plasmid concentrations (measured by Nanodrop spectrophotometer). A standard curve for qPCR was then generated by amplifying the 16S rRNA of the plasmid. The absolute abundance of bacteria in the soil was determined by referring to the standard curve. For fungi, the same method was used to estimate the absolute abundance. Primers (NSIF: 5’-GATTGAATGGCTWAGTGAGG-3’ and 58A2R: 5’-CTGCGTTCTTCATCGAT-3’) [69] targeting the ITS gene were prepared, and *Rhodosporidium* YM25235 was used for amplification of the gene. To analyze the bacteria diversity, the 16S rRNA V3-V4 region was amplified with PCR (95°C for 2 min, followed by 27 cycles at 98°C for 10 s, 62°C for 30 s, and 68°C for 30 s and a final extension at 68°C for 10 min) using the primers 341F: 5’-CCTACGGGNGGCWGCAG-3’ and 806R: 5’-GGACTACHVGGGTATCTAAT-3’, where the barcode is an eight-base sequence unique to each sample. PCRs were performed in triplicate in 50 μL mixtures containing 5 μL of 10 × KOD Buffer, 5 μL of 2.5 mM dNTPs, 1.5 μL of each primer (5 μM), 1 μL of KOD Polymerase, and 100 ng of template DNA. Amplicons were extracted from 2% agarose gels and purified using the AxyPrep DNA Gel Extraction Kit (Axygen Biosciences, Union City, CA, U.S.) according to the manufacturer’s instructions and quantified using QuantiFluor -ST (Promega, U.S.).

Purified amplicons were pooled in equimolar concentrations and paired-end sequenced (2 × 250) on an Illumina platform according to standard protocols. Raw reads were removed if they contained more than 10% of unknown nucleotides (N) or fewer than 80% of bases with quality (Q-value) > 20. Paired-end clean reads were merged as raw tags using FLASH (v 1.2.11) with a minimum overlap of 10 bp and mismatch error rate of 2%. Noisy sequences of raw tags were filtered with the QIIME (V1.9.1) pipeline under specific filtering conditions to obtain high-quality clean tags. Clean tags were searched against the reference database (http://drive5.com/uchime/uchime_download.html) to perform reference-based chimera checking using the UCHIME algorithm (http://www.drive5.com/usearch/manual/uchime_algo.html). All chimeric tags were removed, and the remaining tags were subjected to further analysis (Accession number: PRJNA525653). Tags were clustered into operational taxonomic units (OTUs) of  ≥ 97% similarity using the UPARSE pipeline. The tag sequence with the highest abundance was selected as a representative sequence within each cluster. The representative sequences were classified into organisms with a naive Bayesian model using the RDP classifier (version 2.2) based on the SILVA database (https://www.arb-silva.de/). Shannon index values were calculated in QIIME, out OTU rarefaction and rank abundance curves were also plotted in QIIME. Unweighted UniFrac distance matrices generated by QIIME were used to calculate the beta diversity and were visualized with principal coordinates analysis (PCoA). To identify the bacterial taxa that most likely explained differences among sites, we used the linear discriminant analysis (LDA) effect size (LEfSe) method (http://huttenhower.sph.harvard.edu/galaxy/).

For fungal diversity analysis, the primers ITS3_KYO2F: 5’-GATGAAGAACGYAGYRAA-3’ and ITS4R: 5’-TCCTCCGCTTATTGATATGC-3’, where the barcode is an eight-base sequence unique to each sample, were used. The sequencing procedures were the same as those for the actinobacterial diversity analysis (Accession number: PRJNA525653). The effective tags were clustered into operational taxonomic units (OTUs) of ≥ 97% similarity using UPARSE pipeline. The tag sequence with highest abundance was selected as reprehensive sequence within each cluster. The representative sequences were classified into organisms by a naive Bayesian model using RDP classifier (Version 2.2) based on UNITE Database (https://unite.ut.ee/). Shannon index values were calculated in QIIME, out OTU rarefaction and rank abundance curves were also plotted in QIIME. Unweighted UniFrac distance matrices generated by QIIME were used to calculate the beta diversity and were visualized with principal coordinates analysis (PCoA). The abundance statistics of each taxonomy was construction in a Perl script. Then, the functional group (guild) of the OTUs was inferred using FUNGuild (v1.0). Specifically, FUNGuild can divide the fungi into three types (Pathotroph, Saprotroph and Symbiotroph) according to the published studies. In our study, we used FUNGuild to identify the putative pathogenic fungi of the ant.

### Microorganism isolation and identification

For actinobacterial isolation, 35 soil samples from the nearby the nesting area were collected (S1 Fig). Then 10 g soil of each sample was added to 90 mL of sterile water and shaken for 20 min. Then, 1 mL of liquid was added to 9 mL of sterile water and diluted to concentrations of 10^−5^ and 10^−6^. A 200 μL volume of diluted liquid was then coated onto a plate (Gauze’s Medium No.1) and cultured for 7 days. Colonies with the morphology of actinobacteria were selected for subculturing. Pure cultures were inoculated in Luria-Bertani liquid media, and the liquid cultures were stored in a 25% glycerol solution at -80°C. A Bacterial Genome DNA Extraction Kit (Tiangen, Beijing, China) was used to extract the DNA of the bacteria according to the manufacturer’s instructions. Universal primers (F: 5’-AGAGTTTCATCCTGGCTCAG-3’ and R: 5’-TACGGTTAXXTTGTTACGACTT-3’) were used to amplify the 16S rRNA. The total PCR volume was 50 μL, including 0.4 μL of DNA polymerase (5 U/μL), 5 μL of 10× PCR buffer, 4 μL of dNTPs (2.5 mM), 1 μL of each primer (10 μM), 3 μL of DNA template and 33.6 μL of ddH_2_O. An Eppendorf thermal cycler was used for PCR amplification, and the amplification procedure was as follows: 5 min incubation at 95°C followed by 35 cycles of 95°C for 1 min, 55°C for 1 min and 72°C for 2 min, and a final extension at 72°C for 10 min. The PCR products were confirmed by electrophoresis on a 0.8% agarose gel, and the target PCR product was sequenced (sequencing data were submitted to Figshare, https://figshare.com/articles/bacteria_and_fungi/7370435). The 16S rRNA sequence was sent for BLAST in NCBI (https://blast.ncbi.nlm.nih.gov/Blast.cgi). Based on the hits from the BLAST search, a phylogenetic tree of the identified bacteria was generated with MEGA 6.0 software. The maximum likelihood method was used to construct a phylogenetic tree based on the 16S rRNA sequences, and the phylogenetic tree was evaluated with bootstrap analysis.

For fungal identification, the queens that died in the soil from non-favored nesting areas were collected and cultivated on wet filter papers until fungi grew out from their bodies. Then, the fungi were selected for subculturing on a potato dextrose agar (PDA) plate. Pure cultures were inoculated in potato dextrose broth media, and the liquid cultures were stored in a 25% glycerol solution at -80°C. To identify the fungi, the universal primers of the ITS gene (F: 5’-TCCGTAGGTGAACCTGCGG-3’ and R: 5’- TCCTCCGCTTATTGATATGC-3’) were used. The other procedures were the same as those used for actinobacterial identification (sequencing data were submitted to Figshare, https://figshare.com/articles/bacteria_and_fungi/7370435). To confirm whether the isolated fungi were pathogenic to the queens, the fungi were used to reinfect the queens.

### Quantification of *Streptomyces* and *Nocardiopsis* absolute abundance

qPCR was used to estimate the differences in the absolute abundance of *Streptomyces* and *Nocardiopsis*. For *Streptomyces*, primers (StrepB: 5’-ACAAGCCCTGGAAACGGGGT-3’; StrepF: 5’-ACGTGTGCAGCCCAAGACA-3’) [70] targeting the 16S rRNA gene were prepared, and the genomic DNA of strain 1 was extracted for amplification with the primers. The amplified fragment was then cloned into the pMD 18–T vector, which was then transferred into *E*. *coli* DH5α to reproduce. The reproduced vector was then extracted with a plasmid extraction kit, and the plasmid was diluted in a series of 10-fold dilutions to obtain 5 different plasmid concentrations. A standard curve for qPCR was then generated by amplifying the 16S rRNA of the plasmid. The absolute abundance of *Streptomyces* in the soil was determined by referring to the standard curve. For *Nocardiopsis*, the same method was used to estimate the absolute abundance. Primers (Nsp2: 5’-TCTCTTGGGGTTGACGGTAG-3’; Nsp1: 5’-TAAATGACCTCACATCTCT-3’) [71] targeting the 16S rRNA gene were prepared, and the genomic DNA of strain 3 was extracted for amplification with the primers.

### Antifungal activity of actinobacteria

The inhibitory effect of the isolated actinobacteria on the entomopathogenic fungi (*Aspergillus* sp., *Beauveria* sp. and *Metarhizium* sp.) was tested on PDA plates. Square plugs containing fungi (0.6 cm on one side) were placed in the middle of the plates, and the actinobacterial plugs were placed at the four corners of the square. Then, the plates were cultured for 6 days in the incubator at 28°C. Five replicate plates were done for each fungal taxon and the diameters of fungal growth was compared between treatment and control.

### Survival of queens in different soils with fungal or actinobacterial additions

To test the survival of newly mated queens in soil from favored and non-favored nesting areas, one queen was placed into a plastic square container (length×width×height: 10 cm×10 cm×10 cm) filled with 200 g of newly collected soil from the favored or non-favored nesting areas. Then, the dead queens were recorded and removed after 5, 10, 15, 20, 25 and 30 days. 120 queens were tested in the experiment (60 for favored nesting area, 60 for non-favored nesting area).

For survival tests after fungal infection, the isolated fungi were cultured in Petri dishes (9 cm diam.) containing PDA medium. The fungi were incubated in a constant-temperature incubator at 25 ± 2°C and 75 ± 5(%) RH for 10 days. The conidia were then brushed from the medium and suspended in a 0.01% aqueous solution of Tween-80. The concentration of the solution was measured using a hemocytometer. Conidial preparations were adjusted to 1*10^7^ conidia/mL for the experiments. Then, newly mated queens were submerged in the conidial suspension for 30 s. Ants were then removed from the solution and allowed to walk freely on filter papers for 10 min to remove surface liquid before being placed back in a plastic square container (one queen in one container). Control ants were submerged in a 0.01% Tween-80 solution. Then, the dead queens were recorded and removed after 2, 4, 6, 8 and 10 days.

For survival tests in soil after the addition of the isolated actinobacteria, square plugs of actinobacteria (0.6 cm on one side) were placed in the middle of the plates (Gauze’s Medium No.1), and the plates were cultured for two weeks. Then, 10 mL of sterile water was used to wash off the bacteria, which were evenly added to 200 g of soil collected from a non-favored nesting area. Soil samples containing 10 mL sterile water were used as controls. The soil was kept at room temperature for 3 days, following which 60 newly mated queens were placed into the soil (one queen in 200 g of prepared soil), and the dead queens were recorded and removed after 5, 10, 15, 20, 25 and 30 days. Moreover, the queens that died in the experiments were collected and cultivated on wet filter papers until fungi grew out from their bodies. Then, the fungi were selected for sub culturing on a potato dextrose agar (PDA) plate. Pure cultures were inoculated in potato dextrose broth media and identified using the universal primers of the ITS gene (F: 5’-TCCGTAGGTGAACCTGCGG-3’ and R: 5’- TCCTCCGCTTATTGATATGC-3’).

### Field sampling to test the association between actinobacteria cues and S. invicta nesting behavior

To further confirm the association between actinobacteria cues and *S*. *invicta* nesting behavior, soil samples were collected from another two sites where the ant nests were preferentially distributed (Site 1: N22.9788308914, E113.5229975678; Site 2: N23.176601685121355; E113.42199115344239). For each site, six soil samples (15 cm below the surface) were collected from both favored and non-favored nesting areas. Then the queen choice preference between soil samples collected from favored and non-favored nesting areas were tested as described above. Moreover, volatile contents, *Streptomyces* and *Nocardiopsis* absolute abundance were compared between favored and non-favored nesting areas for the selected two sites as described above.

### Statistical analysis

Attraction of different soils to the queens or workers were determined with Kendall's W, which can be used to test the consistency of multiple related samples. A chi-square test was used to analyze the selective preference of workers or queens in the Y-shape olfactometer. Other the results for different groups were analyzed using independent sample t-test. Survival statistics were calculated using a log-rank analysis and the Kaplan-Meier test. SPSS 19.0 (IBM Corporation, USA) was used for analysis. Graphs were made using Graph-Pad Prism 8.0 (Graph-Pad Software, La Jolla, CA, USA).

## Supporting information

S1 Fig Location of *S*.*invicta* colonies in the study area in Guangzhou, China. The picture was taken by an unmanned aerial vehicle. Each detected ant colony is shown with a red dot. Non-nesting areas are indicated by red rectangles. Red pentagrams indicate the soil sampling sites.
(TIF)

S2 Fig Bacteria diversity in soil collected from favored and non-favored nesting areas. (A), (B) and (C) OTU number and Shannon diversity index of bacteria and actinobacteria in soil (±SE, *n* = 6 biological replicates). (D) PCoA of actinobacteria community in each soil sample. (E) Absolute bacteria abundance in soil achieved by qPCR. Asterisks indicate significant differences (**P < 0.01). NS indicates no significance.
(TIF)

S3 Fig Fungal diversity in soil collected from favored and non-favored nesting areas. (A) and (B) OTU number and Shannon diversity index of fungi in soil (±SE, *n* = 6 biological replicates). (C) PCoA of fungal communities in each soil sample. (D) Absolute fungal abundance in soil. Asterisks indicate significant differences (*P < 0.05). NS indicates no significance.
(TIF)

S1 Data Classification of the OTUs that were considered to be pathogenic. (XLSX)
