# Supplementary figures and images for: The nesting preference of an invasive ant is associated with the cues produced by actinobacteria in soil
Source: PLoS Pathog. 2020 Sep 10;16(9):e1008800. doi: 10.1371/journal.ppat.1008800 (PMC7482974; doi:10.1371/journal.ppat.1008800)

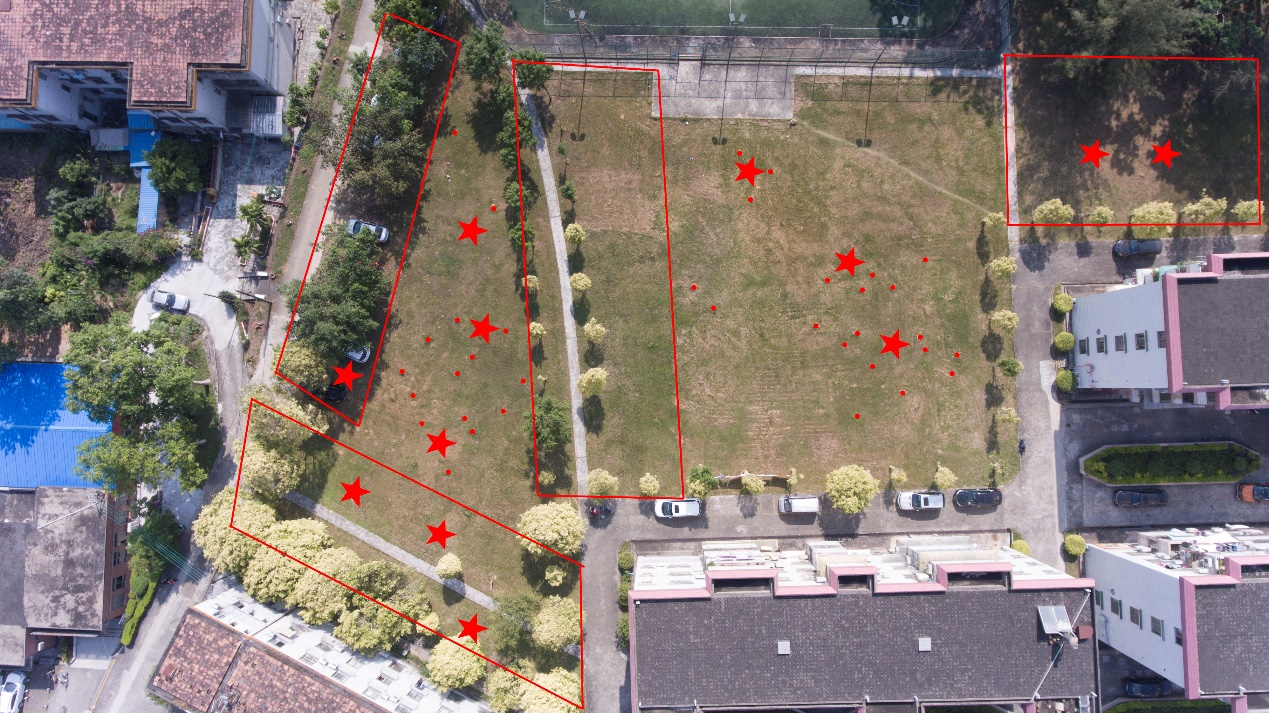

Supplement: S1 Fig — The picture was taken by an unmanned aerial vehicle. Each detected ant colony is shown with a red dot. Non-nesting areas are indicated by red rectangles. Red pentagrams indicate the soil sampling sites. (TIF) [file ppat.1008800.s001.tif]

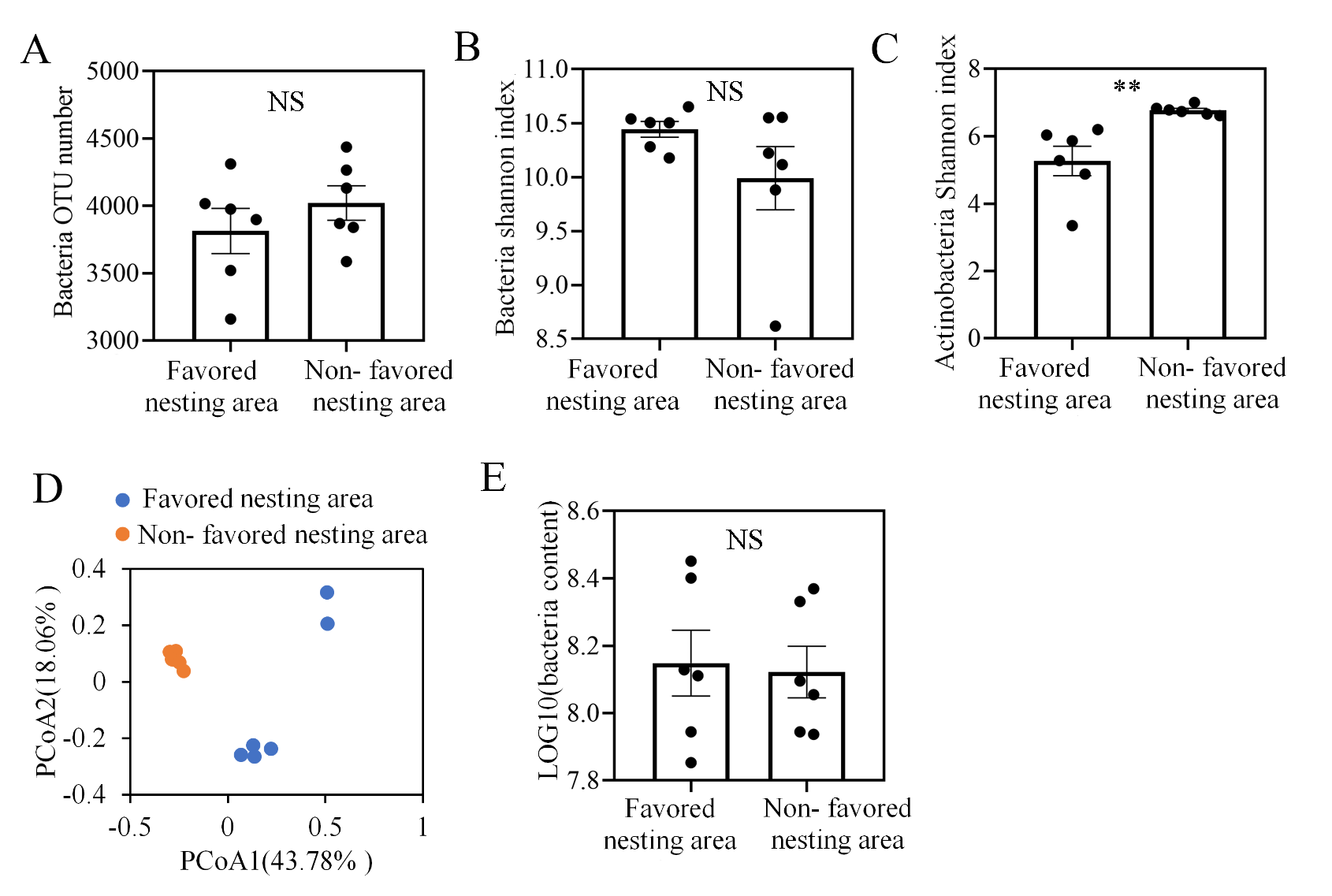

Supplement: S2 Fig — (A), (B) and (C) OTU number and Shannon diversity index of bacteria and actinobacteria in soil (±SE, n = 6 biological replicates). (D) PCoA of actinobacteria community in each soil sample. (E) Absolute bacteria abundance in soil achieved by qPCR. Asterisks indicate significant differences (**P < 0.01). NS indicates no significance. (TIF) [file ppat.1008800.s002.tif]

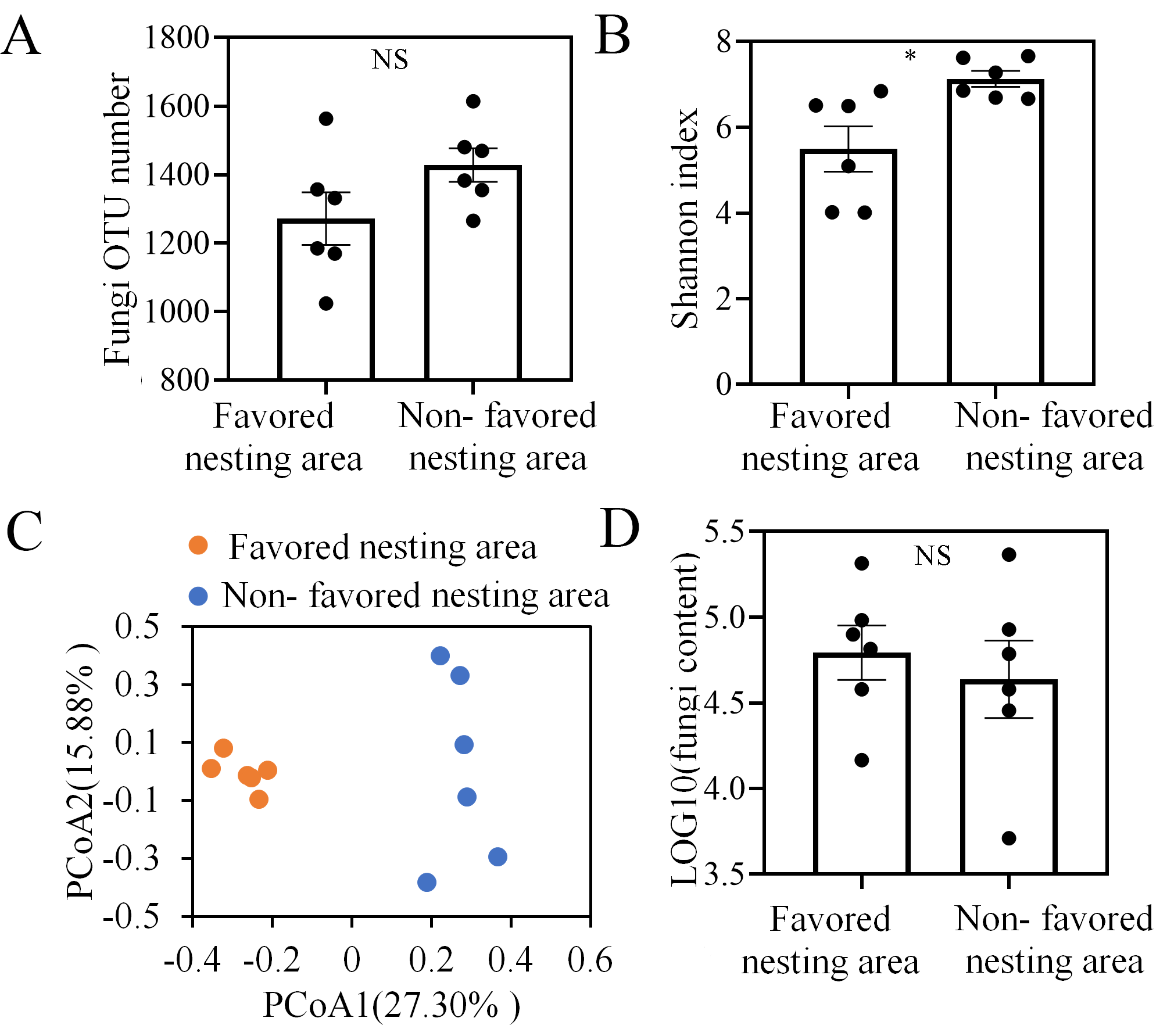

Supplement: S3 Fig — (A) and (B) OTU number and Shannon diversity index of fungi in soil (±SE, n = 6 biological replicates). (C) PCoA of fungal communities in each soil sample. (D) Absolute fungal abundance in soil. Asterisks indicate significant differences (*P < 0.05). NS indicates no significance. (TIF) [file ppat.1008800.s003.tif]
